# Evaluation of Xpert MTB/RIF testing for rapid diagnosis of childhood pulmonary tuberculosis in children by Xpert MTB/RIF testing of stool samples in a low resource setting

**DOI:** 10.1186/s13104-017-2806-3

**Published:** 2017-09-08

**Authors:** Zahra Hasan, Sadia Shakoor, Fehmina Arif, Aisha Mehnaz, Alnoor Akber, Marium Haider, Akbar Kanji, Rumina Hasan

**Affiliations:** 1 Department of Pathology and Laboratory Medicine, The Aga Khan University, Stadium Road, P.O. Box 3500, Karachi, 74800 Pakistan; 2 Department of Pediatrics, Dow University of Health Sciences, Civil Hospital, Karachi, Pakistan

## Abstract

**Objective:**

Children with tuberculosis (TB) remain underdiagnosed due to difficulty in testing for *Mycobacterium tuberculosis* (MTB) infection. We evaluated the Xpert MTB/RIF assay for respiratory and stool testing in children for pulmonary TB through a cross-sectional study at tertiary care facilities in Karachi, Pakistan. Fifty children aged 0–15 years screened by a modified Kenneth-Jones (KJ) score were included. Mycobacterial culture of respiratory samples was the microbiological standard against stool Xpert TB results. All positive TB cases were compared against a treatment response standard (TRS).

**Results:**

Twelve study subjects were diagnosed by Xpert TB and nine by MTB culture. Compared with culture [gastric aspirates (GA)/sputum (spm)], stool Xpert TB had a sensitivity of 88.9% (95% CI 50.7–99.4) and a specificity of 95% (95% CI 81.8–99.1). Xpert TB stool versus GA/spm had sensitivity of 81.8% (95% CI 47.8–96.8) and specificity of 94.7% (95% CI 84.6–99.9). We found good agreement (kappa scores of >0.8) between stool Xpert, GA/spm Xpert and GA/spm culture. Stool Xpert PPV and NPV against TRS was 100 and 82.1% respectively. Stool Xpert TB is a relatively easy option for diagnosis for pulmonary childhood TB in a high burden low-resource setting.

**Electronic supplementary material:**

The online version of this article (doi:10.1186/s13104-017-2806-3) contains supplementary material, which is available to authorized users.

## Introduction

Pakistan ranks 5th amongst high tuberculosis (TB) burden countries and 36% of its population comprises children under 15 years. At least 1 million children fall ill with TB each year. Children represent about 11% of all TB cases [1]. The incidence of TB in Pakistan is 255/100,000 in adults and 46/100,000 in children [1]. Under-diagnosis and under-reporting of TB occurs in children. Diagnosis is often based on clinical signs with algorithms of exposure to TB and chest X-ray (CXR) and immunological reactivity [2, 3]. Microbiological confirmation of TB in young children is difficult due to the problems in obtaining appropriate respiratory specimens; young children do not produce sputum and it is difficult to collect invasive specimens such as gastric aspirates (GA).

Xpert MTB/RIF assay (Cepheid Sunnyvale, USA) allows simultaneous detection of *Mycobacterium tuberculosis* (MTB) and rifampicin resistance, with a pooled sensitivity of 89% for pulmonary TB in adults [4]. In children, the pooled sensitivities and specificities of Xpert for TB detection using respiratory specimens and GA compared with culture, was 62–66 and 98%, respectively [5]. Nicol et al. describe the effectiveness of Xpert stool testing in children compared with induced sputum and GA with a sensitivity of overall 47%, with 80% in HIV infected and 33% in non-HIV infected children [6]. Here, we investigated stool-based Xpert as alternate to respiratory samples from non-HIV children suspected of pulmonary TB.

## Main text

### Methods

The study was conducted at Civil Hospital Karachi (CHK), Dow University of Health Sciences (DUHS), and The Aga Khan University Hospital (AKUH), Karachi, Pakistan (October 2014 to November 2015). CHK is a tertiary care public hospital with pediatric units including, a TB clinic linked with the National Tuberculosis Control Program Pakistan (NTP). The AKUH-Pakistan is a tertiary care teaching hospital. This was a cross-sectional study with convenience sampling method.

Children up to 15 years of age suspected to have pulmonary tuberculosis were screened for the study based on clinical symptoms including, history of fever, cough, weight loss and abdominal distention. Subsequently, a physical exam and chest Xray were performed. Patients were further assessed according to the diagnostic algorithm utilized by the Pakistan Pediatric Association which is based on the NTP case management deskguide [3]. This is a modified Kenneth-Jones (KJ) criteria (Additional file 1: Table S1) [2] and scores patients according to their ‘risk’ assessment based on the presence of risk factors such as, contact with an adult TB patient, lack of BCG vaccination, the occurrence of measles infection, PCM grade III and immune compromise due to malignancies or steroid treatment. We enrolled patients with a KJ score of ≥5 subject to informed consent. One respiratory and stool sample was collected per child. Gastric aspirates (GA) were collected early morning nasogastric intubation following an overnight fast. GA samples were tested by Xpert TB and MTB culture and sensitivity (MTBCS). In patients who produced sputum (spm) years this specimen was utilized instead of GA for Xpert TB and MTBCS.

GA samples collected at CHK were transported to AKUH and processed within 24 h. GA samples were divided into two parts; one was neutralized with 4% sodium carbonate, digested-decontaminated with N-acetyl-L-cystine and 4% sodium hydroxide, centrifuged at 3000 g to set up MTB culture using liquid mycobacterial growth indicator tube (MGIT) 960 system (Becton–Dickinson, USA). Mycobacterial culture and drug susceptibility testing (DST) were performed as described previously [7]. The second part of the respiratory sample was diluted 2:1 in Xpert sample reagent, shaken vigorously, incubated at room temperature for 15 min and added to the Xpert cartridge.

Stool was collected within 24 h of GA samples, stored at 2–8 °C, transported to AKUH and stored at −80 °C until processed as described previously [6]. Briefly, 0.15 g of thawed stool was placed in 2.4 mL phosphate buffered saline (PBS) and vortexed, left undisturbed for 20 min at room temperature before a 1 mL supernatant was removed, centrifuged at 3500 rpm for 15 min. The supernatant was discarded and pellet re-suspended in 1 mL PBS, then diluted 2:1 in sample reagent and added to the Xpert cartridge. Xpert GA results were reported to physicians within 24 h of processing.

Study subjects were diagnosed with TB based on a positive microbiological test result (a positive MTB culture and/or positive Xpert MTB/RIF on gastric aspirate). In addition, some were given empirical treatment based on the evaluation by the treating pediatrician. TB patients were treated according to the Pakistan Pediatric Association guidelines for TB; 2 months of isoniazid (H), rifampicin (R), pyrazinamide (Z) and ethambutol (E) followed with 4 months HR. All patients were followed by regularly every 4–6 weeks and underwent history taking and physical examination. Children were evaluated for adherence to treatment response to treatment (resolution of symptoms, weight gain, radiographic improvement) and monitored for adverse reaction to medications. End of treatment follow up was conducted either in clinic or over the telephone for out of town study subjects.

Patient demographics, Xpert TB and MTBCS results were entered in MS Excel. Xpert stool and Xpert respiratory sample sensitivities were derived by comparing to respiratory culture. FDA Statistical guidance on reporting results from studies evaluating diagnostic tests was used to calculate sensitivity and specificity of the assay and positive and negative percent agreements (PPA, NPA) with kappa scores [8].

Response to empiric anti-tuberculous treatment (ATT) in children who tested negative for microbiological diagnosis (negative TB culture and negative Xpert) was considered as evidence for TB [9, 10]. We used this criterion as a Treatment Response Standard (TRS) to determine the positive predictive value (PPV) and negative predictive value (NPV) and specificity of Xpert in stool. Data was analysed and parameters calculated in MS Excel. [8].

### Results

**Fig. 1:**
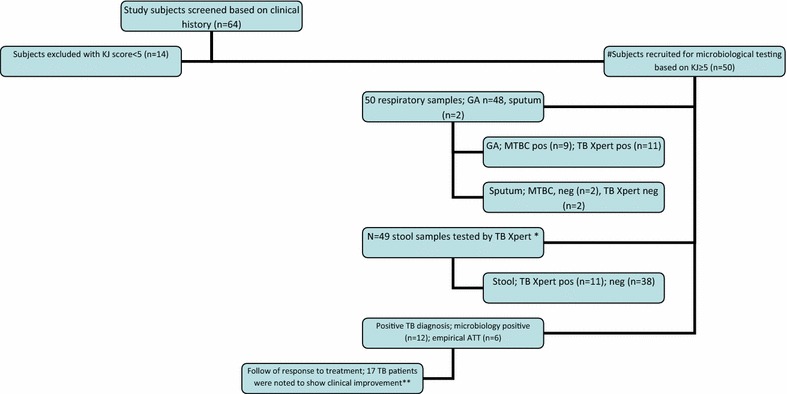
Outline of study. ‘#’ additional tests done for this study together with routine GA/sputum MTB culture were TB Xpert on GA/sputum and stool specimens of each study subject; *asterisk* one study subject expired prior to collection of stool sample; *double asterisk* end of treatment follow up was available for 17 cases only as one study subject expired

A total of sixty-four children were assessed of which fifty were found to fit the study criteria with a KJ score of ≥5, (Fig. 1). Study subjects included 28 males and 22 females (Table 1 and Additional file 2: Table S2) and ages ranging from 7 months to 15 years. The Median (Interquartile range) age of the children was 6.8 (9–2) years. Fifty-four percent (54%; n = 27) of child TB subjects had contact with an adult TB patient. Only 46% (n = 23) of children had a history of BCG vaccination based on parental recall and 30% (n = 15) had a BCG scar. Two children had a history of measles in the 6 months preceding the presenting illness. Forty-six percent (n = 23) of the total group and 61% (n = 14) of under 5 year olds suffered from protein calorie malnutrition (PCM) grade 3. Thirty percent (n = 15) of the overall group and 39% (n = 9) of the under 5 year group were immune-compromised due to steroid treatment, immune-deficiencies or malignancies. Human immunodeficiency virus (HIV) testing was not performed in the study subjects.

**Table 1:** Characteristics of study subjects tested for MTB by Xpert TB/RIF assay or MTB culture

Age group	Number each age group	% of each age group (n = 50)	TB contact (%)	BCG vaccination (%)	BCG scar (%)	Measles (%)	PCM III (%)	Immune compromised (%)	GA Xpert (n = 49)^a^		GA MTBCS (n = 49)^a^		STOOL Xpert (n = 49)^a^	
									Pos	% Pos	Pos	% Pos	Pos	% Pos
0–5 years	23	46	12 (52)	11 (48)	5 (22)	1 (4)	14 (61)	9 (39)	7	31.8	5	22.7	5	27.3
6–10 years	19	38	11 (58)	10 (53)	7 (37)	1 (5)	6 (32)	2 (11)	3	15.7	3	15	2	10
11–15 years	8	16	5 (63)	2 (25)	3 (38)	–	3 (38)	4 (50)	2	25	1	14.3	3	50
Total	50	100	28 (56)	23 (46)	15 (30)	2 (4)	23 (46)	15 (30)	11	22.4	9	18.3	10	20.4

^a^Subjects for whom both GA and stool analysis were available were included

In total 48 GA and 2 spm samples were collected. Stool samples were collected in 49 cases and could not be collected in one case where the subject had expired. Paired respiratory (GA/spm) and Stool samples were available for 49 cases described in Additional file 2: Table S2. All MTB cultures were drug susceptible and similarly, rifampicin resistance was not detected in Xpert positive samples. Microbiological testing of GA samples showed 11 positive cases by Xpert and 9 positive cases by MTBC. The 2 sputum samples were negative by both Xpert and MTBC. Stool Xpert testing revealed 10 positive cases. Table 1 shows results of Xpert GA and stool testing using Xpert and GA MTB culture amongst study subjects.

Overall, 12 children were diagnosed with TB based on microbiological tests and an additional 6 children were diagnosed clinically. All of the eighteen TB cases were prescribed empirical first-line ATT. One child died after 1 month of ATT due to an unrelated accident. The remaining 17 TB cases showed clinical improvement.

When compared with GA culture standard, Xpert in stool had a sensitivity of 88.9% (95% CI 50.7–99.4) and a specificity of 95% (95% CI 81.8–99.1), Table 2.

**Table 2:** Performance characteristics of Xpert in stool in comparison with culture and gastric aspirate Xpert standards

Test	Reference standard	Number of patients included in evaluation	Sensitivity % (95% CI)	Specificity % (95% CI)	PPA (%)	NPA (%)	Kappa agreement (95% CI)
Stool Xpert	GA culture	49	88.9 (50.7–99.4)	95 (81.8–99.1)	88.9	95	0.804 (0.592–1.00)
GA Xpert		49	100 (62.9–100)	95 (81.8–99.1)	100	95	0.875 (0.706–1.00)
GA Xpert	Stool Xpert	49	81.8 (47.8–96.8)	97.4 (84.6–99.9)	81.8	97.3	0.818 (0.62–1.00)

Sensitivity, specificity, 95% confidence intervals (CI), positive percent agreement (PPA), and negative percent agreement (NPA), and kappa scores were calculated in MS Excel

Xpert GA versus culture had sensitivity of 100% (95% CI 62.9–100) and specificity of 95% (95% CI 81.8–99.1). Xpert stool vs GA had sensitivity 81.8% (95% CI 47.8–96.8) and specificity 94.7% (95% CI 80.9–99.1). There was very good agreement (kappa scores of >0.8) between stool Xpert and GA Xpert and GA culture.

Sensitivity, specificity, PPV, and NPV of GA and stool Xpert were also calculated with a treatment response standard (TRS). Xpert in stool had a sensitivity of 58.8% (95% CI 33.5–80.6), specificity of 100% (86.7–100), PPV of 100% (95% CI 65.5–100) and NPV of 82.1% (95% CI 65.9–91.9) against a TRS. Overall percent positive and negative agreements of stool Xpert with GA culture & GA Xpert and Cohen’s kappa is calculated and presented as part of Table 2.

### Discussion

There is a high burden of largely underdiagnosed childhood TB in South Asian countries including, Pakistan [11]. There is a need for rapid, easy diagnostics for child TB in such settings. National guidelines for TB care in Pakistan recommend the use of Xpert as an initial test for diagnosis TB in children. However, due the difficulty in obtaining suitable specimens for testing diagnosis of TB in children has generally been low [12].

Here, we show that Xpert testing of stool has comparable performance and good agreement with Xpert GA testing in a cohort of children with high probability of pulmonary TB. Our data is in line with previous reports [6], including a recent systematic review of Xpert-based childhood TB diagnosis with pooled sensitivity of 62% for respiratory specimens (5).

We observed the sensitivity of stool Xpert to be 88.9% as compared with GA culture, 100% with GA Xpert and 81.8% with GA Xpert. The stool Xpert sensitivity we observed was higher than that shown by Nicol et al. in non-HIV children [6] and by Chipinduro et al. in HIV and non-HIV children [13] possibly, due to the screening tool (modified KJ score) we used to recruit children with high likelihood of TB. However, the sensitivity of Xpert stool we observed was similar to that reported by Banada et al. [14] who tested larger quantities of replicate stool samples from children with pediatric TB. A limitation of our study was that we were able to only collect one set of stool and GA samples. Marcy et al. showed that when additional respiratory samples such as, nasopharyngeal aspirates were taken together with stool Xpert the sensitivity was increased by 10%, improving chances of TB diagnosis [15].

The NTP has installed more than 50 Xpert machines installed in selected tertiary, secondary care hospitals and specialized TB clinics. As stool is easily obtained we hope that this work can be extended to implementation in the NTP, allowing the described method to be implemented in a decentralized setting. It would be most effective to combine stool Xpert with testing of additional respiratory samples (GA, NPA) by Xpert for the diagnosis of childhood TB as it is highly likely to improve yield of TB diagnosis. Overall, we show that stool Xpert testing can improve microbiological diagnosis and confirmation of TB in children at high risk for TB in the local setting.

### Limitations

Due to the limited study budget the sample size of the study was relatively small. Also, we were only able to collect a single stool sample rather than successive daily samples. HIV testing was not performed on the study subjects.

## Additional files

**Additional file 1: Table S1.** Modified Kenneth Jones Score/ Pakistan Paediatrics Association scoring chart for diagnosis of TB in children. ‘*0-2’ TB unlikely; ‘3-4’ Keep under observation for possible TB for 3 months; ‘5-6’ Tuberculosis possible (Investigations may justify therapy; ‘7’ or more TB probable and needs to be confirmed.* ‘*’ Include children with malignancies (leukemias, lymphomas), immunodeficiencies, and immunosuppressive therapy such as chronic steroids more than 2 weeks. PCM Grade 3= Protein Calorie Malnutrition grade 3 not improving after 4 weeks of adequate caloric intake. ‘**’ Physical Examination Suggestive of TB: Pulmonary findings (unilateral wheeze, dullness), hepatosplenomegaly, ascites; Strongly suggestive of TB: Matted lymphadenopathy, abdominal mass, gibbus formation, chronic monoarthritis, CNS findings (bulging fontanelle, irritability, papilledema). ‘†’ Radiological findings: Nonspecific: Ill-defined opacity/infiltrates; marked broncho-vascular marking. Suggestive of TB: Consolidation not responding to antibiotic therapy; paratracheal, tracheal, or mediastinal lymphadenopathy, miliary mottling.

**Additional file 2: Table S2.** Characteristics of Pediatric Study subjects enrolled for TB testing. Data was compiled as per information collected from Modified Kenneth Jones Score sheet (Additional file 1: Table S1). The presence of an identifier is, “1” and absence is, “0”; na, not available.

## Abbreviations

Xpert TB: Xpert MTB/RIF assay, Cepheid, USA
TB: tuberculosis
MTB: *Mycobacterium tuberculosis*
KJ: Kenneth-Jones
GA: gastric aspirates
Spm: sputum
TRS: treatment response standard
PPV: positive predictive value
NPV: negative predictive value
CXR: chest X-ray
CHK: Civil Hospital Karachi
DUHS: Dow University of Health Sciences
AKUH: The Aga Khan University Hospital
NTP: National Tuberculosis Control Program Pakistan
MGIT: Mycobacterial Growth Indicator Tube
DST: drug susceptibility testing
PBS: phosphate buffered saline
PPA: positive percent agreement
NPA: negative percent agreements
ATT: anti-tuberculous treatment
PCM: protein calorie malnutrition
HIV: human immunodeficiency virus
