# Supplementary material for: Evaluation of Xpert MTB/RIF testing for rapid diagnosis of childhood pulmonary tuberculosis in children by Xpert MTB/RIF testing of stool samples in a low resource setting
Source: BMC Res Notes. 2017 Sep 8;10:473. doi: 10.1186/s13104-017-2806-3 (PMC5591572; doi:10.1186/s13104-017-2806-3)
Supplement: Supplementary file 1 — Additional file 1: Table S1. Modified Kenneth Jones Score/ Pakistan Paediatrics Association scoring chart for diagnosis of TB in children. ‘0-2’ TB unlikely; ‘3-4’ Keep under observation for possible TB for 3 months; ‘5-6’ Tuberculosis possible (Investigations may justify therapy; ‘7’ or more TB probable and needs to be confirmed. ‘*’ Include children with malignancies (leukemias, lymphomas), immunodeficiencies, and immunosuppressive therapy such as chronic steroids more than 2 weeks. PCM Grade 3= Protein Calorie Malnutrition grade 3 not improving after 4 weeks of adequate caloric intake. ‘**’ Physical Examination Suggestive of TB: Pulmonary findings (unilateral wheeze, dullness), hepatosplenomegaly, ascites; Strongly suggestive of TB: Matted lymphadenopathy, abdominal mass, gibbus formation, chronic monoarthritis, CNS findings (bulging fontanelle, irritability, papilledema). ‘†’ Radiological findings: Nonspecific: Ill-defined opacity/infiltrates; marked broncho-vascular marking. Suggestive of TB: Consolidation not responding to antibiotic therapy; paratracheal, tracheal, or mediastinal lymphadenopathy, miliary mottling. [file 13104_2017_2806_MOESM1_ESM.doc]

**Supplementary Table 1: Modified Kenneth Jones Score/ Pakistan Paediatrics Association scoring chart for diagnosis of TB in children**

**Please circle as appropriate for the scoring**

| Features | | | | | | |
| --- | --- | --- | --- | --- | --- | --- |
| **HISTORY** | | | | | | |
| **Features** | 1 | 2 | 3 | 4 | 5 | **Score** |
| Age | <2 years | - | - |  | - |  |
| Contact in last 2 years | with TB patient |  | With sputum+ve TB patient |  |  |  |
| BCG Scar | Absent | - |  |  |  |  |
| History of measles and whooping cough | Between 3-6 months | < 3 months |  |  |  |  |
| Immunocompromised/ immunosuppressed* | Yes | - |  |  |  |  |
| PCM Grade 3 | Yes | - | Not improving |  |  |  |
| **EXAMINATION AND INVESTIGATION** | | | | | | |
| Physical examination** | - | Suggestive of TB |  | Strongly suggestive |  |  |
| Radiological findings† | Non specific | Suggestive of TB |  |  |  |  |
| Tuberculin skin test | 5-10 mm |  | >10mm |  |  |  |
| Granuloma | Non specific |  |  |  | Specific for TB |  |
| **TOTAL SCORE** | | | | | | |
