# Supplementary material for: Evaluation of Xpert MTB/RIF testing for rapid diagnosis of childhood pulmonary tuberculosis in children by Xpert MTB/RIF testing of stool samples in a low resource setting
Source: BMC Res Notes. 2017 Sep 8;10:473. doi: 10.1186/s13104-017-2806-3 (PMC5591572; doi:10.1186/s13104-017-2806-3)
Supplement: Supplementary file 2 — Additional file 2: Table S2. Characteristics of Pediatric Study subjects enrolled for TB testing. Data was compiled as per information collected from Modified Kenneth Jones Score sheet (Additional file 1: Table S1). The presence of an identifier is, “1” and absence is, “0”; na, not available. [file 13104_2017_2806_MOESM2_ESM.doc]

**Supplementary** Table 2. Characteristics of Pediatric Study subjects enrolled for TB testing

| **No.** | **Age range (y)** | **Sex** |  | **Data from modified Kenneth-Jones score** | | | | | | | **Xpert** | | **AFBC** |
| --- | --- | --- | --- | --- | --- | --- | --- | --- | --- | --- | --- | --- | --- |
|  |  |  | **KJS** | **BCG vaccine** | **TB contact** | **Chest Xray finding** | **BCG scar** | **measles history** | **PCM grade 3** | **Immuno-comp/supp.** | **GA** | **Stool** | **GA** |
| 1 | 0-5 | M | 6 | 0 | 1 | 1 | 0 | 0 | 0 | 0 | 0 | 0 | 0 |
| 2 | 0-5 | F | 7 | 1 | 0 | 1 | 1 | 0 | 1 | 1 | 0 | 0 | 0 |
| 3 | 11-15 | M | 7 | 0 | 1 | 1 | 0 | 0 | 0 | 0 | 0 | 0 | 0 |
| 4 | 0-5 | M | 14 | 0 | 1 | 1 | 0 | 0 | 1 | 1 | 1 | 1 | 1 |
| 5 | 11-15 | M | 6 | 0 | 1 | 0 | 1 | 0 | 0 | 0 | 0 | 0 | 0 |
| 6 | 0-5 | M | 5 | 0 | 1 | 1 | 0 | 0 | 0 | 0 | 0 | 0 | 0 |
| 7 | 6-10 | F | 6 | n/a | n/a | 1 | 0 | 0 | 1 | 0 | 0 | 0 | 0 |
| 8 | 6-10 | M | 6 | 0 | 0 | 1 | 0 | 0 | 1 | 1 | 0 | 0 | 0 |
| 9 | 0-5 | M | 5 | 1 | 1 | 1 | 0 | 0 | 0 | 0 | 0 | 0 | 0 |
| 10 | 6-10 | M | 5 | 1 | 1 | 1 | 1 | 0 | 0 | 0 | 0 | 0 | 0 |
| 11 | 6-10 | M | 10 | 0 | 1 | 0 | 0 | 0 | 1 | 0 | 0 | 0 | 0 |
| 12 | 0-5 | F | 7 | n/a | 1 | 1 | 0 | 0 | 1 | 1 | 1 | 0 | 0 |
| 13 | 6-10 | M | 6 | 1 | 0 | 1 | 0 | 0 | 0 | 0 | 0 | 0 | 0 |
| 14 | 6-10 | F | 5 | 1 | 0 | 1 | 0 | 0 | 0 | 0 | 0 | 0 | 0 |
| 15 | 6-10 | M | 8 | 0 | n/a | 1 | 0 | 0 | 0 | 0 | 0 | 0 | 0 |
| 16 | 0-5 | F | 7 | 1 | 1 | 1 | 0 | 0 | 1 | 0 | 0 | 0 | 0 |
| 17 | 11-15 | F | 6 | 0 | 1 | 1 | 0 | 0 | 0 | 0 | 1 | 1 | 1 |
| 18 | 0-5 | F | 10 | 0 | 0 | 1 | 0 | 1 | 1 | 1 | 0 | 0 | 0 |
| 19 | 0-5 | F | 9 | 1 | 0 | 1 | 0 | 0 | 1 | 1 | 1 | 1 | 1 |
| 20 | 0-5 | F | 8 | n/a | 1 | n/a | 1 | 0 | 1 | 1 | 0 | 0 | 0 |
| 21 | 11-15 | M | 7 | n/a | 1 | 1 | 1 | 0 | 1 | 1 | 1 | 1 | 1 |
| 22 | 6-10 | M | 5 | 1 | 1 | 1 | 1 | 0 | 0 | 0 | 0 | 0 | 0 |
| 23 | 0-5 | F | 8 | 0 | 1 | 1 | 0 | 0 | 1 | 0 | 0 | 0 | 0 |
| 24 | 0-5 | M | 7 | 1 | 1 | 1 | 0 | 0 | 1 | 1 | 0 | na | 0 |
| 25 | 11-15 | M | 6 | 0 | 0 | 1 | 0 | 0 | 1 | 0 | 0 | 0 | 0 |
| 26 | 0-5 | F | 9 | 1 | 0 | 1 | 0 | 0 | 1 | 1 | 1 | 1 | 1 |
| 27 | 0-5 | F | 5 | 1 | 0 | 1 | 0 | 0 | 1 | 0 | 0 | 0 | 0 |
| 28 | 0-5 | F | 8 | 0 | 1 | 1 | 0 | 0 | 0 | 0 | 1 | 1 | 1 |
| 29 | 0-5 | M | 10 | 1 | 0 | 1 | 0 | 0 | 0 | 0 | 0 | 0 | 0 |
| 30 | 6-10 | F | 7 | 0 | 1 | 0 | 0 | 0 | 1 | 0 | 0 | 0 | 0 |
| 31 | 6-10 | F | 7 | 1 | 1 | 1 | 1 | 0 | 0 | 0 | 0 | 0 | 0 |
| 32 | 6-10 | M | 6 | 0 | 1 | 1 | 0 | 0 | 0 | 0 | 0 | 0 | 0 |
| 33 | 0-5 | F | 5 | 0 | 1 | 1 | 0 | 0 | 0 | 0 | 0 | 0 | 0 |
| 34 | 0-5 | M | 10 | 1 | 0 | 1 | 0 | 0 | 1 | 0 | 0 | 0 | 0 |
| 35 | 6-10 | M | 6 | 1 | 0 | 1 | 1 | 0 | 1 | 1 | 0 | 0 | 0 |
| 36 | 11-15 | F | 6 | 1 | 0 | 1 | 1 | 0 | 1 | 1 | 0 | 0 | 0 |
| 37 | 6-10 | F | 7 | 1 | 1 | 1 | 0 | 1 | 0 | 0 | 1 | 1 | 1 |
| 38 | 6-10 | F | 8 | 1 | 0 | 1 | 0 | 0 | 0 | 0 | 1 | 0 | 1 |
| 39 | 6-10 | M | 6 | 1 | n/a | 1 | 0 | 0 | 0 | 0 | 0 | 0 | 0 |
| 40 | 0-5 | M | 6 | 0 | n/a | 1 | 0 | 0 | 0 | 0 | 1 | 1 | 1 |
| 41 | 6-10 | M | 5 | n/a | 1 | 1 | 1 | 0 | 0 | 0 | 0 | 0 | 0 |
| 42 | 6-10 | M | 7 | 1 | 1 | 1 | 1 | 0 | 0 | 0 | 0 | 0 | 0 |
| 43 | 11-15 | F | 8 | 1 | 1 | 1 | 0 | 0 | 0 | 0 | 0 | 0 | 0 |
| 44 | 11-15 | F | 6 | 0 | n/a | 1 | 1 | 0 | 1 | 1 | 0 | 1 | 0 |
| 45 | 0-5 | M | 5 | 1 | 0 | 1 | 1 | 0 | 0 | 0 | 0 | 0 | 0 |
| 46 | 0-5 | M | 8 | 0 | 0 | 1 | 1 | 0 | 0 | 0 | 0 | 0 | 0 |
| 47 | 11-15 | M | 6 | 0 | 0 | 1 | 0 | 0 | 0 | 1 | 1 | 1 | 0 |
| 48 | 0-5 | M | 8 | 1 | 0 | 1 | 1 | 0 | 1 | 1 | 0 | 0 | 0 |
| 49 | 11-15 | F | 10 | 0 | 1 | 1 | 0 | 0 | 0 | 0 | 0 | 0 | 0 |
| 50 | 0-5 | M | 5 | 0 | 1 | 1 | 0 | 0 | 1 | 0 | 0 | 0 | 0 |
|  |  |  |  |  |  |  |  |  |  |  |  |  |  |
